# Health without Medicine

**Published:** 1889-08

**Authors:** Theodore N. Mead


### HEALTH WITHOUT MEDICINE.

BY THEODORE N. MEAD.

No. 2.

My stay in England was protracted some two months after leaving Brighton, and I occupied the time in little journeyings hither and thither spending the Sundays, whenever possible, in the delightful Cathedral cities. Dumb bells, Indian Clubs and such gymnastic appliances were manifestly impossible under the circumstances, so I arranged a series of exercises which might be carried on in any bedroom, without noise or jar, and with a few modifications they have served me ever since. Nobody who has not actually tried the experiment, knows how great a bore such exercises are, and how almost irresistible the temptation to omit them now and then, and that too, with the conviction that such omission is only the first step toward total neglect. To diminish in some degree the tiresomeness of them, it occurred to me to make a systematic effort to increase my personal symetry by developing those muscles, and there were plenty of them, which were conspicuously deficient. My success in this effort I will confess to the sympathetic reader, has given me a pleasure which it is to be feared, might be considered childish by some of my friends, but which has certainly made perseverance much easier to me. For economy of time I have found it best in the morning to do part of the work before my bath, and in the intervals of preparation for it, and part of it afterward while dressing. In this way my toilet occupies from the time of getting up till I sit down to breakfast just about an hour ; sometimes more, sometimes less, and I avoid setting the heart beating too violently, as well as getting in too profuse a perspiration.

The exercises I go through with, are the following which I take in the order as given.

Before beginning each exercise, stand perfectly straight with the breast thrown forward and draw a deep breath. Observe that the clothing should be loose and of some light woolen material.

1. Stretch she arms out straight in front, horizontally, with clenched fists turned palm downward, and swing them up and down vigorously, at the same time moving them gradually apart without bending the elbow, till they are as far back as possible. Begin with ten times and increase gradually to forty six times up and down with each arm.

This builds up the muscle on the top of the shoulder, and is therefore an exercise useful to men and boys with too sloping shoulders, but it will naturally be seldom made use of by women.

2. Stand erect and spring up off the floor, straightening the leg and changing at each spring the position of the feet by placing first one in advance and then the other. Alight as noiselessly as possible. At first ten times increasing gradually to forty-six times for each foot.

This exercise is especially good for the calves, and therefore equally advantageous for men and women. My own practice is to do it at the same time as No. 1.

3. Stretch the arms out in front as in No. 1. and swing them horizontally backward and forward, bringing them forward as swiftly as possible, as if to strike them together but stopping them when the hands are about six inches apart. Beginning with ten, increase gradually to forty, six times.

This developes the muscle of the breast between the ribs and shoulder filling up the ungraceful hollows too often seen there, while at the same time expanding the chest, and it is therefore especially to be recommended to those who are inclined to be round shouldered.

4. Draw the fists up in front of the shoulders, and strike out as forcibly as possible, first with one and then with the other. Begin with ten and increase to forty-six times with each.

This while working the pectoral muscles, especially developes those at the back of the upper arm and will therefore recommend itself to the ladies.

5. Stand erect and kick each foot alternately up behind as high and as quickly as possible. Begin with ten and increase gradually to forty-six times with each foot.

This developes especially the calves and the back of the thigh, greatly increasing the symmetry of the leg.

My practice is to do it at the same time as No. 4, a combination which will be found to set the blood in active circulation with great promptness.

6 Stand firmly on the left foot, step forward about a yard with the right, keeping the left knee straight, but bending the right so that the leg below will be vertical, precisely as when making a lunge in fencing. Now lean forward, touch both hands to the floor and raise them, without bending the elbows, as high above the head as possible, touch them again to the floor, and resume your upright position. Now do the same stepping forward with the left foot and keeping the right knee unbent. Begin with three times each and increase to ten times on each foot.

This is a sort of universal strengthener, and if well and energetically done with the proper muscular tension, will have a powerful effect on back, loin and abdomen as well as on legs and arms. When at home I use dumb bells of sixteen pounds each for this exercise, diminishing the number to five times on each foot.

7. Stand erect and swing the arms swiftly and alternately in circles, striking out as forcibly as possible with each fist at a point about forty-five degrees above the level of the shoulder. To make this exercise at once profitable and graceful, the circles should be true and in the same vertical plane—that is, the arms should not be thrown careless about, but pass close in front of the person. Begin with twenty times and increase to seventy with each arm. When at home I use long and slender Indian clubs of three pounds weight each, in which case I swing them each forty-six times. For children I would recommend clubs of one pound, and for ladies no heavier, but longer ones.

8. The same as No. 7, but reversed. Swing each arm in the opposite direction, striking out at a point about forty-five degrees below the level of the shoulder. Begin with twenty times and increase to seventy, or with Indian clubs to forty-six.

9. Swing the clubs as in No. 8, but behind the back, striking out so as to straighten the arm in the same manner. Begin with five and increase to twenty times each.

10. Swing the clubs as in No. 9, but behind the back. Begin with two and increase to ten each.

All the exercises with the clubs should be especial favorites with the ladies, as they round the arms and shoulders and fill out the upper part of the breast, while they have a most beneficial influence on the whole upper part of the body.

11. Stand erect with the feet close together, raise the open hands as high as possible, then bow downward, with the head between the arms, without bending the knees, touch the fingers to the floor, or come as near to it as you can. Do this at first, once, and increase to five times. I could not reach the floor at all, when I began, but can now lay my open palms upon it.

This exercise is excellent for the muscles of the stomach and bowels, and very developing to those of the back, leg and thigh.

12. Stand firmly on the left foot, and, with the knees unbent, raise the right foot as high as you can and hold it so for a few seconds. Now do the same with the left. Then again raising the right foot carry it around horizontally to the right till it is straight behind, keeping it as high as possible and then bring it back in the same way. Do the same with the left.

These exercises develop the front thigh and train the hip muscles.

13. Sit down on both heels, and, rising suddenly, spring as high as possible into the air, alighting without noise on the toes. At first, twice, finally ten times.

14. Place both hands against the wall, standing some little distance back, and with the knees unbend lean forward till the breast touches it, then push yourself as far back as possible. Begin with three or four times and continue till you can do it ten times, substituting a couple of chairs for the wall.

This is excellent for breast and arm muscles.

15. Stand erect, stretch out the right foot and sit gradually down on the left heel and rise up again without touching the floor with the right foot or with the hands. Do this, at first, once on each foot, gradually increasing to five times on each foot, keeping the lungs inflated, the head up, the shoulders back.

This exercise strengthens the knees and develops the front of the thigh; it has slso a powerful and beneficial effect on the concealed muscles of the loin and abdomen. It had better, however, be begun with both feet, and so continued a considerable time, especially by ladies and persons in delicate health.

(*To be Continued.*)

